# Effects of Natural Health Products in Combination with FP-Based Chemotherapy

**DOI:** 10.3390/ph18111767

**Published:** 2025-11-20

**Authors:** Valeria Conti, Berenice Stefanelli, Carmineantonio Romeo, Alessandra De Stefano, Dominga Valentino, Graziamaria Corbi, Francesco Sabbatino, Emanuela De Bellis, Amelia Filippelli

**Affiliations:** 1 Department of Medicine, Surgery, and Dentistry, Scuola Medica Salernitana, University of Salerno, 84081 Baronissi, Italy; vconti@unisa.it (V.C.); fsabbatino@unisa.it (F.S.); afilippelli@unisa.it (A.F.); 2 Clinical Pharmacology Unit, San Giovanni di Dio e Ruggi d’Aragona University Hospital, 84131 Salerno, Italy; bstefanelli@unisa.it (B.S.); dominga.valentino@studenti.unicampania.it (D.V.); 3 Postgraduate School of Clinical Pharmacology and Toxicology, University of Salerno, 84081 Baronissi, Italy; c.romeo6@studenti.unisa.it (C.R.); a.destefano42@studenti.unisa.it (A.D.S.); 4 Postgraduate School in Clinical Pharmacology and Toxicology, Department of Experimental Medicine, University of Campania “Luigi Vanvitelli”, 80138 Naples, Italy; 5 Department of Translational Medical Sciences, University of Naples “Federico II”, 80131 Naples, Italy; graziamaria.corbi@unina.it; 6 Oncology Unit, University Hospital “San Giovanni di Dio e Ruggi D’Aragona”, 84131 Salerno, Italy; 7 PhD School “Clinical and Translational Oncology (CTO)”, Scuola Superiore Meridionale, University of Naples “Federico II”, 80138 Naples, Italy

**Keywords:** capecitabine, natural health products, dietary supplements, herbal medicine, fluoropyrimidines, interactions, nutritional supplements, folate supplements, folate intake, folic acid intake

## Abstract

**Background:** Cancer patients often use natural health products (NHPs) during chemotherapy without medical supervision. We have previously described the clinical cases of two patients taking capecitabine in combination with folate supplements who suffered from severe diarrhoea and hand-foot syndrome, emphasising that the combination of NHPs with chemotherapeutic agents such as fluoropyrimidines (FPs) can lead to life-threatening events. Although the potential harmful interaction between folate supplements and capecitabine is reported in the summary of product characteristics for this FP, it remains unclear, and evidence regarding interactions with other NHPs is even more limited. **Objectives**/**Methods**: This narrative review aimed to provide an update on the literature regarding the effects of combining NHPs and FPs, describing the results of randomised clinical trials and observational studies to provide a critical analysis of the factors influencing the clinical outcomes of cancer patients following this therapeutic approach. **Results**: Herbal supplements belonging to traditional Chinese medicine and other NHPs, including polyunsaturated fatty acids and probiotics, may reduce the incidence and severity of gastrointestinal, haematological, and skin toxicities related to FPs. In addition to potential safety benefits, NHPs may improve the efficacy of FP-based therapy. Folate supplements appear to improve efficacy outcomes, such as disease-free survival and overall survival, but have also been associated with serious FP-related adverse events. However, the results are mixed, partly because they are influenced by the patient’s genetic background. **Conclusions**: Overall, the available data are inconclusive and do not support the introduction of natural products as complementary therapy in cancer patients undergoing FP-based chemotherapy, highlighting the need for further investigation.

## 1. Introduction

Fluoropyrimidines (FPs), such as 5-fluorouracil (5-FU) and capecitabine (CAP), are widely used in the treatment of various solid malignancies, such as gastrointestinal, breast, and head and neck cancers [1]. 5-FU and CAP are often used in combination with other chemotherapeutic agents, especially oxaliplatin (OX) in FOLFOX and CAPOX regimens and irinotecan (IRI) in FOLFIRI and CAPIRI regimens, respectively.

The main mechanism of action of FPs consists of inhibiting thymidylate synthase (TS) [1]. TS converts deoxyuridylate (dUMP) to deoxythymidylate (dTMP), in which the folate cofactor 5,10-methylenetetrahydrofolate (5,10-MTHF) works as a methyl donor. 5-FdUMP and 5,10-MTHF inhibit TS irreversibly, forming a ternary complex. The lack of intracellular dTMP leads to decreased DNA synthesis, dUMP misincorporation into DNA, and DNA strand breaks followed by cell apoptosis [2]. A secondary mechanism of action involves the incorporation of specific FP metabolites, such as fluorouridine triphosphate (FUTP) into RNA, leading to impaired RNA synthesis and function and contributing to cellular toxicity. Another metabolite, fluorodeoxyuridine triphosphate (FdUTP) can be incorporated into DNA, causing fragmentation and instability and thus contributing to antiproliferative activity [3]. Over 80% of FPs is metabolised by the enzyme dihydropyrimidine dehydrogenase (DPD), encoded by the DPYD gene. DPD converts 5-FU to 5,6-dihydro-5-fluorouracil (DHFU), which is subsequently metabolised by other enzymes (such as dihydropyrimidinase and β-ureidopropionase) into inactive metabolites, such as α-fluoro-β-alanine (FBAL), which are excreted in the urine [4]. Variability in DPD activity affects 5-FU efficacy and toxicity, potentially leading to severe and life-threatening adverse events (AEs). DPYD genotyping helps identify patients at high risk of serious AEs, allowing dosage adjustments or selection of alternative drugs to improve treatment safety [5,6]. In addition, DPD inhibitors, such as uracil and eniluracil, can be administered together with FPs to slow down their degradation, thereby improving therapeutic response rates [3]. Cancer patients, including those treated with FP-based therapy, often combine Natural Health Products (NHPs) to prevent or mitigate chemotherapy-related toxicity, improve their quality of life (QOL) and enhance the effectiveness of drugs [7,8].

The use of NHPs, such as those belonging to traditional Chinese medicine (TCM), dietary supplements, probiotics and vitamins, is recognized as an unconventional therapeutic approach referred to as Complementary and Alternative Medicine (CAM) [9]. NHPs are often self-administered and purchased from unverified online sources, typically without the guidance of a healthcare professional [10]. This is a cause for great concern because NHPs can cause toxic effects on their own and can interact negatively with conventional chemotherapy drugs. In particular, supplements containing folic acid, widely used by cancer patients, have been associated with serious AEs when administered concomitantly with FPs [1,7,11]. This narrative review aimed to give a comprehensive update on the efficacy and safety of NHPs when taken in combination with FP-based chemotherapy, describing the results of randomised clinical trials (RCTs) and observational studies available in the literature over the last two decades to provide a critical analysis of the factors influencing the clinical outcomes of cancer patients following this therapeutic approach.

## 2. Methodology

We conducted a non-systematic review of the literature with the aim of investigating the efficacy and safety outcomes of NHPs administered in combination with FP-based therapy.

Observational studies and RCTs were selected based on a study design that involved the combined use of FP-based chemotherapy regimens with NHPs and discussed clinical outcomes associated with this therapeutic approach. Study quality was assessed using the Jadad scale for RCTs, which assigns a score from 0 to 5 points [12] and the Newcastle–Ottawa scale for observational studies, which assigns a score from 0 to 9 points [13].

PubMed, Cochrane and Scopus databases, and ClinicalTrials.gov were searched using the following keywords: “capecitabine”, “natural health products”, “dietary supplements”, “herbal medicine”, “fluoropyrimidines”, “interactions”, “nutritional supplements”, “folate supplements”, “folate intake”, “folic acid intake”.

## 3. Results

Both RCTs (Table 1) and prospective and retrospective observational studies (Table 2) conducted on cancer patients receiving FP-based chemotherapy regimens in combination with NHPs were examined.

Most of the studies concern NHPs belonging to TCM, followed by polyunsaturated fatty acids (PUFAs), essential amino acids and folate-based supplements.

The patients participating in these studies suffered from various solid malignancies, primarily gastrointestinal cancer (GI) followed by breast, pancreatic, and hepatocellular cancers. All patients received FP-based therapy including CAP as monotherapy [14,15,16,17,18,19,20,21,22,23,24,25,26,27,28] or CAP in combination with OX (CAPOX) [14,19,22,23,24,25,27,29,30,31,32,33,34,35,36,37,38] or 5-FU as monotherapy [17,22,30,36] or 5-FU in combination with OX (FOLFOX) [14,19,22,27,30,32,33,35,38] or with IRI (FOLFIRI) [19,27,33,35] or other regimen containing FPs [39,40].

The following paragraphs are classified in such a way as to describe the effects of NHPs in preventing and/or reducing the incidence and severity of each AE or adverse drug reaction (ADR), focusing on those most frequently attributed to FP-based treatment. A separate section has been devoted to describing the potential role of NHPs in improving the efficacy of chemotherapeutic agents, with a focus on the most important endpoints such as overall survival (OS), progression-free survival (PFS) and disease-free survival (DFS). Finally, the last section describes the effects of combining folate supplements and FPs.

**Table 1:** Main characteristics and results of RCTs investigating the safety of NHPs in cancer patients treated with fluoropyrimidine-based chemotherapy.

Study Design	Country	Patients (n)	Type of Cancer	Sex (F%)	Median Age and Age Range (Years)	Food Supplements	Concomitant Drugs	Follow-Up	Efficacy Outcomes	Main Results and Conclusion	Jadad Score	Ref.
RCT	Iran	46 INT-g: 23 CT-g: 23	CRC	30.5	58.3 (48–68)	Mediterranean diet regime with EVOO	FOLFOX or CAPOX or CAP	NA	Valuation of muscle strength, lean body mass, nutritional status, inflammatory markers, QOL, serum albumin and total protein	A significant increase in weight, lean mass, fat mass, fat percentage, muscle strength and total serum protein was recorded in the INT-g compared to the CT-g (*p* < 0.001). In addition, there was a significant reduction in TNF-α, Hs-CRP and IL-6 in the INT-g group compared to the CT-g group (*p* < 0.001).	3	[14]
RCT	China	52 pz INT-g: 27 CT-g: 25	GC	41	69.3 (65–75)	Elemene (active principle of Chinese herbal medicine *Wenyujin*, member of the ginger family) 0.6 gr	Lobaplatin perfusion (50 mg/m^2^) CapeOx	9.04 ± 4.30 (3.00–17.90)	Incidence of ADR myelosuppression, immune function, average hospital length of stay	Compared to patients in the INT-g, patients in the CT-g showed no difference in the incidence of complications during hospitalisation, but had an increase in length of hospital stay (*p* = 0.045) and severe myelosuppression (*p* = 0.027). Furthermore, the immune function of patients in the INT group was less compromised (*p* < 0.001) than in the CT-g group.	2	[29]
Randomized, placebo-controlled, triple-blind clinical trial	Brazil	56 INT-g (FOG group): 28 CT-g (PG group): 28	GI	47	54.5 (41–63)	EPA and DHA (Omega 3 Concentrate^®^) 1.55 gr/day	CAPOX or OX + FU or FU + LV	6 and 12 months	AEs	No difference between groups in the occurrence of adverse events. However, patients in CT-g showed more severe diarrhoea than patients in INT-g (*p* = 0.03) and a worse performance status score (*p* = 0.02).	5	[30]
Pilot, randomized, triple-blinded, placebo-controlled clinical trial	Iran	110 INT-g: 55 CT-g: 55	CRC	66.7	56.3 (42–68)	Alpha^®^ ointment: combination of *Lawsonia inermis* (Henna, 2-hydroxy-1,4-naphthoquinone) and *Curcuma longa* (curcumin) 3 g and 0.15/30 gr for three weeks	XELOX + LAP	2 months	Alpha^®^ ointment prevention of HFS	No significant difference in HFS prevention (*p >* 0.05) was found between the groups. However, in the intervention group, a delay in the onset of HFS episodes and a worsening of symptoms were observed (*p* < 0.001).	3	[31]
Randomized, double-blinded, placebo-controlled, phase II trial	Japan	100 INT-g: 52 CT-g: 48	CRC	44	65 (20–80)	L-cystine (700 mg), L-theanine (270 mg) and other ingredients (maltitol, aspartame and citric acid) to a total of 1.5 g	CAP 1250 mg/m^2^ twice daily orally for 2 weeks followed by a 1-week’s suspension in a 3-week cycle.	6 months	Incidence of diarrhea of grade 1 or higher, incidence of other AEs	The incidence of grade 1 or higher diarrhoea and grade 1 or higher HFS is lower in the INT-g than in the CT-g, but without reaching statistical significance.	5	[15]
Randomized, double-blinded, placebo-controlled clinical trial	China	370 INT-g: 186 CT-g: 184	CRC	42.7	60.3 (32–75)	PRM1201(*Ligustrum lucidum*, *Cistanche deserticola*, *Iphigenia indica*, *Vitis quinquangularis*, *Panax ginseng*, *Akebia trifoliata*, and *Salvia miltiorrhiza* twice a day for 6 months	FOLFOX or CAPOX	113	ADRs	Grade 3–4 neutropenia, diarrhea, vomiting, and nausea was less recurring in the INT-g than in the CT-g, but without reaching statistical significance.	5	[32]
Double blind, placebo-controlled pilot study	Slovenia	60 INT-g: 30 PL-g: 30	CRC	41	62.1 (46–74)	Glutamine 30 gr t.i.d for 5 weeks	Radiotherapy + oral CAP at a daily dose of 1650 mg/m^2^.	NA	G.I toxicity indicated by diarrhea, variation of inflammatory and metabolic parameters	Glutamine supplementation did not reduce the incidence and severity of diarrhoea and did not affect inflammatory and metabolic activity compared to maltodextrin placebo treatment.	4	[16]
Double-blind, placebo-controlled, randomized phase II study	Japan	90 INT-g: 43 CT-g: 47	CRC	44.2	67 (29–85)	TJ-14 (*Pinelliae tuber*, *Scutellariae Radix*, *Glycyrrhizae Radix*, *Zizyphi Fructus*, *Ginseng Radix*, *Zingiberis Processum rhizoma*, *and Coptidis rhizome*) 2.5 g t.i.d for 14 or 21 days	FOLFOX FOLFIRI or XELOX	Until AEs are resolved or no longer clinically significant	Safety of TJ-14 in reducing the incidence of severe oral mucositis (WHO grade ≥ 3)	Patients in the INT group had a shorter median duration of grade ≥2 mucositis (*p* = 0.018) compared to those treated with placebo and a lower incidence of grade ≥2 oral mucositis compared to CT-g, but without reaching statistical significance.	4	[33]
Pilot, randomized, double-blind, placebo-controlled clinical trial	Iran	18 INT-g: 9 CT-g: 9	CRC	55.6	53.9 (44–73)	Topical hydrogel (H.gel) containing the hydroalcoholic extract (10%) of *Henna* (0.62 mg *Lawsone* in each 100 g) Qid.	CAP or 5-FU	Two weeks	To evaluate the number of participants who experienced at least one grade improvement in HFS based on CTCAE v4.03 at any point during the protocol treatment.	No statistically significant differences were found between the two groups at baseline, after treatment, or in grade variations.	5	[17]
Multi-center, phase III, randomized, placebo-controlled trial	China	400 INT-g: 200 CT-g: 200	CRC	43.1	60.3 (60–80)	JPBS (*Codonopsis pilosula*, *Atractylodes Macrocephala*, *Wolfiporia Cocos*, *Rhizoma Pinelliae*, *Citri Reticulatae Pericarpiu*, *Radix Glycyrrhizae Preparata*, *Astragalus membranaceus*, *Cuscuta chinensis Lam*, *Fructus Ligustri Lucidi*, *Angelicae Sinensis Radix*, *Herba Ecliptae*, *Fructus Psoraleae*)	CAPOX	NA	Chemotherapy-related toxicities, AEs, and SAEs	JPBS decreased the incidence of grade ≥2 vomiting (*p* = 0.007) but led to an increase in grade ≥2 thrombocytopenia (*p* = 0.012).	5	[34]
Multi-institutional randomized-controlled trial	Japan	22 INT-g: 12 CT-g: 10	CRC	59	66.2 (56–79)	Eppikajututo TJ-28 (extract granules from *gypsum*, *ephedra herb*, *Atractylodes lancea*, *jujube*, *glycyrrhiza*, *and ginger*) 7500 mg/day	8 cycles of capecitabine 2500 mg/m^2^ per day for 14 days, followed by a 7-day rest period	Every 3 weeks	Effectiveness of TJ-28 in preventing HFS	TJ-28 did not significantly prevent CAP-associated HFS when compared to pyridoxine.	2	[18]
Multicenter, randomized, double-blindplacebo-controlled clinical trial	China	112 INT-g: 56 PL-g: 56	CRC	39.3	63.1 (52–74)	Hezhong granules (*Pinellia ternata*, *Evodia rutaecarpa*, and *Zingiber officinal*) 6 g three times daily, for a total daily dose of 18 g from days 1–14 of chemotherapy, followed by a 7-day break.	FU-based chemotherapy (CAPEOX, mFOLFOX6, FOLFIRI, and CAP monotherapy (plus 5-HT3-receptor antagonist, DXM)	NA	Efficacy and safety in the prevention of CINV	The INT-g showed statistically significant improvements of approximately 20% for both nausea and vomiting (*p* = 0.053; *p* = 0.035) compared to the CT-g, but only in the delayed phase. In addition, the daily incidence of CINV events showed a mean difference of 19% (*p* < 0.05).	4	[19]
Single-center, randomized double-blind, placebo-controlled phase 3 trial	Singapore	208 INT-g: 104 CT-g: 104	Breast, CRC, and other cancers.	77	58 (26–82)	Pyridoxine (200 mg)	CAP 1000 mg/m^2^ on a 3-weekly cycle (8 cycles)	NA	Incidence and time to onset of grade 2 or greater HFS	The incidence of grade 2 or higher HFS did not differ between groups. No significant differences were found in the severity and incidence of other AEs.	5	[20]
Randomized, multiple-center, double-blind, and parallel-controlled trial	China	156 INT-g: 78 CT-g: 78	CRC	70.4	55.5 (38–72)	Chinese herbal compound LC09 (*Astragalus membranaceus*, *flowers carthami*, *lithospermum*, *geranium wilfordii*, *and radix angelicae*) 110 gr per day	CAP-containing chemotherapy for 2 weeks and then they pause for 1 week	1 month and 3 months after completing the trial	HFS improvement rate (based on NCI grading), Numerical Rating Scale (NRS) pain scores, and pain reduction rate.	After treatment, the INT-g showed a higher effectiveness rate in managing HFS (*p* < 0.01) and greater pain relief compared to the control group (*p* < 0.01).	5	[21]

Abbreviations: ADR, Adverse Drug Reactions; AEs, Adverse Events; B, bevacizumab; CAP, capecitabine; CAPOX, capecitabine/oxaliplatin; CRC, colorectal cancer; CINV, Chemotherapy-Induced Nausea and Vomiting; CTCAE, Common Terminology Criteria for Adverse Events; DHA, docosahexaenoic acid; DOC, Docetaxel/Oxaliplatin/Capecitabine; DXM, dexametasone; EOX, Epirubicin/Oxaliplatin; EPA, eicosapentaenoic acid; EVs, Extracellular vesicles; EVOO, extra virgin olive oil; FJQR, Fuzheng jiedu Quyu Method Recipe; FOLFIRI, fluorouracil/leucovorin/irinotecan; FOLFOX, fluorouracil/leucovorin/oxaliplatin; FU, fluorouracil; FU/LV, fluorouracil/leucovorin; GC, gastric cancer; GI, gastrointestinal cancer; GEF, gefitinib; HFS, Hand-foot syndrome; JPBS JianPi-Bushen Formula; LAP, Lapatinib; LV, leucovorin; PN, Peripheral Neuropathy, PN; NYT, Ninjin’yoeito; ONS, Oral Nutritional Supplements; OX, oxaliplatin; QOL, quality of life; Ref., References; SAEs, serious adverse events; S-1, Tegafur, 5-Chloro-2-4-Dihydroxypyridine and Oxonic Acid; SF, Sorafenib; TJ-14, Hangeshashinto; TNF-ɑ, Tumor Necrosis Factor-alpha; WGJ, wheatgrass juice; WL, Weight loss; XELOX, Capecitabine/Oxaliplatin.

**Table 2:** Main characteristics and results of observational studies investigating the safety of NHPs in cancer patients treated with fluoropyrimidine-based chemotherapy.

Study Design	Country	Type of Cancer	Patients (n)	Sex (F%)	Median Age and Age Range (Years)	Food Supplements	Concomitant Drugs	Follow-Up	Safety Outcomes	Main Results and Conclusion	NOS	Ref.
Not Randomized clinical trial (volunteers bias)	Israel	CRC	100 INT-g: 50 CT-g: 50	44.4	64 (29–83)	WGJ young grass extract of the common wheat plant *Triticum aestivum*, containing: chlorophyll, flavonoids, 17 amino acids, eight of which are essential, vitamins A, C, and E, and high mineral content (iron, calcium, magnesium, zinc)	1. CAP alone or 2. CAP + OX or 3.5-FU/LV + OX	16 months	Difference in chemotherapic effects between CC patients with and without supportive treatment of WGJ	A significant increase in monocyte count (*p* = 0.002), reduction of vascular damage, chemotherapy-related thrombogenicity (levels of tissue factor *p* = 0.029 and endothelial protein C receptor *p* = 0.005), growth factors, cytokines (*p* = 0.00), and endothelial EVs (*p* = 0.0015) were observed in the INT-g group compared to the CT-g group.	6	[22]
Prospective study	China	CRC	126 Low folate-g = 63 High folate-g = 63	50	60 (55–68)	Folate supplements	CAP- or CAPOX-based treatment	Every three weeks	Evaluate whether serum and red-cell folate levels are predictors of CAP-related toxicity.	Serum folate level (*p* = 0.002), but not red-cell folate, was an independent predictor of grade ≥2 CAP-related toxicity.	7	[23]
Phase II single-arm clinical trial with historical CT-g group	United Kingdom	GC	58 INT-g: 21 Historical CT-g: 37	27.6	66.5 (36–81)	Omega-3 fatty acids (Omegaven^®^) once a week for a 4 h infusion once a week	EOX (E 50 mg/m^2^ and OX 130 mg/m^2^ every 21 days and oral CAP)	Minimum of 12 months	Tumour radiological response and toxicity	Grade 3 or 4 toxicity, particularly thromboembolism and GI disorders was less frequently observed in INT-g (*p* = 0.04).	6	[39]
Prospective study	Italy	54 CRC, 16 GC, 6 pancreatic, 1 breast 1 unknown primary cancer	INT-g: 76 CT-g: Historical data	36.8	67 (NA)	*Lactobacillus kefiri LKF01* (Kefibios^®^) 5 drops/day	FP-based treatment (FOLFOX, FLOT, XELOX, FOLFIRI, FOLFIRINOX, FOLFOXIRI)	6 months	Assess the clinical efficacy of Kefibios^®^ in the prevention or treatment of severe diarrhea induced by FPs treatment	Supplementation with Kefibios^®^ has been shown to be effective in the prevention and treatment of severe diarrhea in cancer patients treated with FP.	6	[35]
Cross-sectional study	Malaysia	243 CRC, 61 Breast, 29 GC, 13 Nasopharyngeal, 9 pancreatic, 5 oesophageal, 9 others	369 HFS-g: 185 no-HFS-g: 184	50.4	57 (NA)	folic acid intake	CAP, CAPOX	4.5	Estimate HFS CAP-induced prevalence and impact and risk factors	Significant risk factors for grade ≥ 2 HFS included higher dose of CAP and folic acid intake (*p* = 0.008 and *p* = 0.612, respectively).	8	[24]
Prospective cohort study	Netherlands	CRC	290 Dietary/supplemental intake at diagnosis g = 280/283 Dietary/supplemental intake during chemotherapy-g 262/266 Biomarkers at diagnosis -g: 212 Biomarkers during chemotherapy-g: 221	38	64 (60–69)	Dietary folate intake and folate supplements 10 µg DFE/day	CAPOX, CAP alone: 800–1000 mg/m^2^, administered twice daily for 14 d followed by a 7-d rest period	6	Association between folate intake and biomarkers with toxicities	Folate intake and plasma folate levels were not associated with the risk of toxicity. It should be noted, however, that the use of folic acid supplements during treatment and the presence of folic acid in the plasma at the time of diagnosis were associated with an increased risk of toxicity.	8	[25]
Retrospective study	China	GC	129 INT-g: 64 CT-g: 65	28.7	NA (18–75)	“Fuzheng jiedu Quyu Method” recipe (FJQR) (*Astragalus*, *Pseudostellariae radix*, *Spica Prunellae*, *Curcumae radix*, *Herba Oldenlandiae*). The principal chemical components are *Astragalus* polysaccharides, *Radix Prunella*, *Prunella vulgaris* saponins 15 g Bid	CAP-based treatment (CAP 1000 mg/m 2 per os, Qid)	<1 month	Safety	The incidence of grade III-IV AEs was significantly lower than in the CT-g (*p* < 0.05). No significant difference was found in the incidence of other grades of AEs.	7	[26]
Prospective cohort study	Netherlands	CRC	325 INT-g: 127 CT-g: 193	32.6	67 (58–76)	Multivitamins/multiminerals, folate supplements, herbal supplements	5-FU, CAPOX	6 weeks–6, 12 and 24 months	Association between supplement use with fatigue	No statistically significant differences in fatigue scores over time were observed between groups. In the interindividual analysis, INT-g showed greater fatigue than CT-g.	8	[36]
A single-arm phase II clinical trial with historical cohort	USA	CRC	24	25	54.5 (50–62)	YIV-906 *Glycyrrhiza uralensis Fisch (G)*, *Paeonia lactiflora Pall (P)*, *Scutellaria baicalensis Georgi (S)*, *and Ziziphus jujuba Mill (Z)* 800 mg bid on days 1–4 of RT each week	CAP (orally at a dose of 825 mg/m 2 bid, on days 1–5 of RT (pelvic radiation therapy) each week or FOLFIRI or CAPOX or FOLFOX	61.9	Reduction in GI side effects	Adding YIV-906 to CAP-based chemoradiation resulted in lower rates of GI toxicity compared to historical controls, particularly for grade 3 or higher diarrhea.	6	[27]
Retrospective study	China	GC	100 INT-g: 50 CT-g: 50	51	55.5 (48–63)	Zhipu Liujunzi decoction *(Panax ginseng*, *Polygonatum sibiricum*, *Scrophularia ningpoensis*, *Ganoderma sinense*, *Smilax glabra*, *Cremastra appendiculata* and *Bambusa tuldoides*, *Astragalus mongholicus*, *Atractylodes macrocephala*, *Amomum villosum*, *Citrus medica* var. *sarcodactylis*, *Citrus reticulata*, *Pinellia ternata*, *Zingiber officinale*) These herbs all were decocted in water to be oral administered once a daily for 2 continuous weeks.	OX 130 mg/m^2^ e.v b.i.d once a day and orally taking 1500 mg of CAP b.i.d for consecutive 14 day	1 month	Evaluate the clinical safety of co-administration of Zhipu Liujunzi decoction with CAPOX treatment	INT-g demonstrated lower rate of toxicities than the CT-g (*p* < 0.05).	6	[37]
Retrospective study	China	GC	115 INT-g: 57 CT-g: 58	31	54.8 (48–62)	Shengbai decoction (*Radix Astragali* 10 g, *Radix Salviae Miltiorrhizae* 10 g, *Ganoderma Lucidumseu Sinensis* 6 g, *Rhizoma Atractylodis Macrocephalae* 10 g, *Radix Angelicae Sinensis* 10 g, *Caulis Spatholobi* 15 g, *Fructus Ligustri Lucidi* 10 g, *Rhizoma Polygonati* 10 g, *Fructus Psoraleae* 10 g and *Herba Dendrobii* 10 g) 100 mL twice a day half an hour before meals	SOX, CAPOX, FOLFOX	Every 3 months	Effect of Shengbai decoction on the improvement of myelosuppression induced by chemotherapy	Patients who took Shengbai decoction tolerated the therapy better, with fewer hematological ADRs than the CT-g (*p* < 0.05).	7	[38]
Multicenter, open-label, dose escalation phase I/II safety and efficacy clinical trial	USA	Hepato cellular	42 Phase I: 18 Phase II: 24	51	NA (18–80)	PHY906 (consists of a mixture of four herbs: (*Scutellaria baicalensis Georgi*, *Glycyrrhiza uralensis Fisch.*, *Paeonia lactiflora Pall.*, *and the fruit of Ziziphus jujube Mill.*) (600, 800, or 1000 mg *bid*) was administered orally on days 1–4 and 8–11 of each 21-day course	CAP 750 or 1000 mg/m^2^ bid was administered orally starting on day 1, and continued for 14 consecutive days followed by 7 days’ rest	For all toxicity duration, documentation of disease progression and OS	Toxicity, and QoL	The combination of PHY906 and capecitabine was well tolerated and AEs were not related to CAP/PHY906 administration.	5	[28]
Clinical trial	China	GC	60 INT-g: 30 CT-g: 30	NA	NA (18–75)	Kanglaite^®^ Injection (Coix Seed Oil) 100 mL intravenous per day during chemotherapy	DOC	Every three weeks	Side effects of Kanglaite^®^ injection	GI side effects and myelosuppression were lower in INT-g than in CT-g (*p* < 0.05).	6	[40]

Abbreviations: 5-FU, 5-Fluorouracil; ADR, Adverse Drug Reactions; AE(s), Adverse Event(s); BID, Bis in die (twice daily); BJOEI, Brucea javanica Oil Emulsion Injection; CAP, Capecitabine; CAPOX, Capecitabine + Oxaliplatin; CRC, Colorectal cancer; CT-g, Control Group; DOC, Docetaxel, Oxaliplatin, Capecitabine; E, Epirubicin; EOX, Epirubicin + Oxaliplatin + Capecitabine; EVs, Extracellular Vesicles; FPs, Fluoropyrimidines; FJQR, Fuzheng Jiedu Quyu Recipe; FLOT, 5-FU + Leucovorin + Oxaliplatin + Docetaxel; FOLFIRI, 5-FU + Leucovorin + Irinotecan; FOLFOX, 5-FU + Leucovorin + Oxaliplatin; FOLFIRINOX, 5-FU + Leucovorin + Irinotecan + Oxaliplatin; FOLFOXIRI, 5-FU + Leucovorin + Irinotecan + Oxaliplatin; GC, gastric cancer; HFS, Hand-Foot Syndrome; INT-g, Intervention Group; LV, Leucovorin; NA, Not Available; NOS, Newcastle–Ottawa scale; OX, Oxaliplatin; PD, Progressive Disease; PHY906/YIV-906, Herbal compound including Scutellaria, Glycyrrhiza, Ziziphus, and Paeonia; Ref., References; RT, Radiotherapy; SOX, S-1 + Oxaliplatin; WGJ, Wheatgrass Juice.

### 3.1. Role of NHPs in Contrasting Fluoropyrimidine-Induced Gastrointestinal Disorders

FPs can cause severe and even fatal toxicity in 10–40% of patients, especially when combined with other chemotherapy drugs. Gastrointestinal AEs are very common, particularly diarrhoea, nausea and vomiting. Diarrhoea has been reported in up to 50% of patients undergoing weekly treatment with 5-FU/leucovorin (LV), and the severity of this AE may increase when 5-FU is administered as a bolus rather than a continuous infusion [41]. However, factors such as gender and race, as well as polymorphisms in dihydropyrimidine dehydrogenase (DPYD) and other genes involved in the FP pathway, influence clinical outcome [6,42]. The most commonly used NHPs belong to TCM and consist of a wide range of substances of plant, mineral, or animal origin that have been used as part of the Chinese medical system for thousands of years [43,44]. TCM products are often combinations of different types of herbs that have been proven to be stable and clearly effective and have therefore been documented and passed down as formulas through the generations. This has led to the development of over 300,000 known formulas that form the basis of clinical treatments in TCM [45]. Zhou et al. investigated the potential role of PRM1201, a formula based on seven medicinal herbs. Through in vitro and in vivo experiments, these authors demonstrated that PRM1201 is capable of inhibiting the invasion process and metastasis production in colorectal cancer (CRC) patients [46,47].

Subsequently, a RCT was conducted on 370 patients who were randomised to receive FOLFOX or CAPOX plus PRM1201 or the same chemotherapy regimens with placebo. It was observed that the incidence of grade 3–4 diarrhoea, as well as nausea and vomiting, tended to be lower than in patients receiving placebo [32]. Another typical herbal formula, called JianPi-Bushen (JPBS), has been widely used for 20 years to reduce the severity of chemotherapy-related toxicity in patients with CRC, particularly gastrointestinal disorders. The improvement in gastrointestinal symptoms associated with JPBS appears to be due to the modulation of c-myc expression, which leads to a reduction in proinflammatory cytokine levels, particularly involved in regulating the microbiome and protecting against gastrointestinal lesions [48]. The results of this pre-clinical study were confirmed by a phase 3 randomised double-blind study conducted in 13 Chinese hospitals between 2018 and 2021 on 400 patients randomised to receive CAPOX + JPBS twice daily or (CAPOX + placebo). The difference in the incidence of grade 2 vomiting was statistically significant (3.8% in the JPBS arm vs. 6.4% in the placebo arm, *p* = 0.007). No differences were observed in the incidence of nausea, diarrhoea and constipation. Conversely, the incidence of grade 2 thrombocytopenia was significantly higher in the JPBS arm than in the placebo arm (16.2% vs. 12.4%, *p* = 0.012) [34]. Hezhong granules, a traditional Chinese herbal formula containing *Pinellia ternata* (Thunb.), *Evodia rutaecarpa* (Juss.), and *Zingiber officinale*, are widely used to prevent chemotherapy-induced nausea and vomiting (CINV) and have recently shown promising anti-tumor activity. To date, no specific preclinical studies have been conducted on Hezhong granules to date, with the exception of Banxiaxiexin decoction and Wuzhuyu decoction, which have been shown to alleviate symptoms of nausea and vomiting and reduce gastrointestinal reactions in rats, rabbits and minks [49,50]. These latter formulations share several components with Hezhong granules. Wu et al. conducted a multicentre randomised clinical trial involving 112 CRC patients who were randomised in a 1:1 ratio to receive FP-based chemotherapy combined with conventional antiemetic therapy comprising a 5-HT3 antagonist and dexamethasone plus Hezhong granules or placebo. In the acute phase (defined as CINV occurring within 24 h), an improvement, although not statistically significant, was observed in nausea and vomiting in the experimental group, while a significant improvement compared to placebo was observed in the complete response rate (CCR), (*p* = 0.053 for nausea and *p* = 0.035 for vomiting) and the objective response rate (ORR) after 16 months of NHP intake (*p* = 0.037 for nausea and *p* = 0.043 for vomiting). Furthermore, the daily frequency of these AEs was 19% lower in the intervention group than in the control group (*p* < 0.05) [19]. Kanglaite^®^ (Coix seed oil) is one of the most widely used Chinese herbal preparations for the treatment of non-small cell lung cancer, liver cancer and gastric cancer [40,51]. In a study conducted by Zhan et al., 60 patients undergoing the DOC regimen, consisting of docetaxel, OX and CAP and intravenous injection of Kanglaite^®^, were randomised 1:1 with the control arm consisting of patients treated with DOC alone. The experimental group showed a significant reduction in the incidence of diarrhoea, nausea and vomiting compared to the control group (*p* < 0.05) [40].

Promising results have been obtained with the use of N-3 polyunsaturated fatty acids contained in fish oil, mainly eicosapentaenoic acid (EPA) and docosahexaenoic acid (DHA). Since these molecules have several double bonds, they are highly peroxydable and more susceptible to attack by reactive oxygen species and the onset of lipid peroxidation. The increase in the production of compounds derived from lipid peroxidation, which in turn increases susceptibility to cell death, has been linked to a reduction in tumor size [52,53]. Furthermore, omega-3s have been shown to exert a synergistic effect with chemotherapy, improving drug absorption and modulating certain intracellular targets, including COX-2, NFKB, PPAR-γ, MAPKs, AKT, and BCL-2/BAX [54]. The efficacy and toxicity of EPA and DHA in combination with FP-based chemotherapy compared to a control consisting of a supplement containing extra virgin olive oil was evaluated in a randomised, triple-blind study conducted by De Quadros Camargo et al. After 9 weeks of treatment, the overall incidence of AEs did not differ between groups. However, the severity of diarrhoea was significantly lower in the fish oil-treated group (*p* = 0.03). No significant difference in hematological toxicity was observed [30]. Eltweri et al. conducted a single-arm, non-randomised study with a historical control group in 21 patients with advanced esophago-gastric adenocarcinoma undergoing palliative chemotherapy (epirubicin/OX/CAP, EOX) combined with weekly infusion of Omegaven^®^ (a fish oil–based emulsion, rich in omega-3 FA used in parenteral nutrition). A significant reduction in nausea/vomiting (*p* = 0.04) and thromboembolic events (*p* = 0.04) was observed in the group treated with EOX plus fish oil. Conversely, the percentage of patients with grade 3 or 4 neutropenia and leukopenia was higher in the group receiving EOX plus fish oil than in the group receiving EOX alone (*p* = 0.002 and *p* < 0.001, respectively) [39].

Glutamine is the most abundant amino acid in humans and is an essential nutrient for the immune system, as it is a source of energy for immune cells and a fundamental amino acid necessary for their differentiation and growth [55,56]. Malignancies and their treatment generate a systemic inflammatory response and metabolic distress, thus affecting the bioavailability of glutamine for immunocytes [57]. In vivo studies demonstrated that a glutamine supplementation before or after total body irradiation reduced the severity of acute and chronic radiation-related toxic effects, including mucosal lesions, inflammation, and intestinal dysfunction [58,59]. On the other hand, in a double-blind, placebo-controlled pilot study conducted by Kozjek et al., 60 patients with CRC were randomised in a 1:1 ratio to an experimental arm using 30 g of glutamine 3 times daily for 5 weeks with radiotherapy and CAP and a control arm receiving radiotherapy plus CAP in combination with maltodextrin. It was observed that the incidence and severity of toxicity, particularly diarrhoea, were not inferior to the control group. Furthermore, glutamine did not appear to affect the metabolic and immune activity of patients [16].

Thanks to their anti-inflammatory properties, probiotics are also able to suppress intestinal inflammation through the downregulation of Toll-like receptor expression and through the secretion of molecules capable of inhibiting the TNF-α and NF-κB pathways in enterocytes. Through these mechanisms, probiotics restore the intestinal flora and provide benefits in the treatment of gastrointestinal diseases, including recurrent C. difficile-induced diarrhoea and inflammatory bowel diseases. In addition, they may be useful in patients with chemotherapy-related diarrhoea [60,61].

In particular, kefir grains are characterised by a rich symbiotic microbial complex with the presence of *Lactobacillus kefiri* (LK). In an in vivo study, in which LK was administered daily to healthy mice for 21 days, an increase in IgA in faeces and reduced expression of pro-inflammatory mediators in Peyer’s patches and mesenteric lymph nodes was observed, with a concomitant increase in IL-10 levels [62]. When administered to healthy volunteers, LK contributed to the rebalancing of the gut microbiota by reducing the presence of several bacterial genera involved in the onset of pro-inflammatory responses and gastrointestinal diseases [63]. In a prospective observational study, 76 patients (mainly with CRC and gastro-oesophageal cancers) undergoing both adjuvant and palliative therapy received 5 drops per day of Kefibios^®^, whose main strain was LK, in combination with FP-based chemotherapy. Probiotic intake was shown to reduce significantly the incidence of grade 3–4 diarrhoea to 3.9% in the 1st and 2nd cycles, with a consequent sharp decrease from the 3rd cycle onwards (1.3%) and complete resolution of this symptom from the 5th cycle onwards compared to the use of chemotherapy regimens alone, where the rate of grade 3–4 diarrhoea ranged from 10 to 20% depending on the drugs used in the chemotherapy regimen [35].

In preclinical studies in mice, YIV-906 (or PHY906), a Chinese herbal medicine formulation composed of four herbs (*Glycyrrhiza uralensis Fisch*, *Paeonia lactiflora Pall*, *Scutellaria baicalensis Georgi*, and *Ziziphus jujuba Mill*), has been shown to reduce damage to the intestinal mucosa by decreasing the infiltration of inflammatory cells and, consequently, the expression of various pro-inflammatory cytokines after treatment with a wide range of chemotherapeutic agents (5-FU, CAP, IRI, and gemcitabine). Furthermore, YIV-906 is able to activate the Wnt (Wingless and Int-1) signalling pathway in gastrointestinal tract stem cells, contributing to the protection of the intestinal epithelium from cytotoxic damage and reducing morphological changes caused by radiation, including flattening and loss of villi, as well as fluid loss and crypt hyperplasia [64]. In phase I clinical trials on CRC and hepatocellular cancer, YIV-906 was found to reduce the incidence of grade 3 diarrhoea without altering the pharmacokinetics of the coadministered chemotherapy drugs [28,65]. A single-arm phase 2 clinical trial conducted by Verma et al., confirmed the results of in vivo studies and preliminary clinical investigations. In 24 patients with rectal adenocarcinoma, YIV-906 (800 mg, twice daily) was used in combination with radiotherapy and CAP-based treatment. A 62-month follow-up demonstrated that the combination of this NHP with CAP-based chemoradiotherapy resulted in lower rates of gastrointestinal toxicity compared to historical controls, particularly with regard to grade ≥ 3 diarrhoea [27]. Another multicentre, open-label, phase I/II study with progressive dose escalation evaluated the safety and efficacy of PHY906 administered concomitantly with CAP in patients with advanced hepatocellular carcinoma. While phase I was conducted primarily to determine a safe and tolerable dosing regimen of PHY906 plus CAP, phase II was designed to evaluate whether this NHP improved the response rate to CAP and the onset of CAP-related ADRs. The PHY906/CAP combination caused grade 3 toxicity in only six patients among the twenty-seven evaluable for efficacy, and no related grade 4 or 5 toxic events were observed. The rate of nausea and vomiting with the combination was lower than would normally be expected with CAP alone. In addition, only two patients discontinued the combination treatment due to AEs compared with CAP alone (5.1% vs. approximately 29%) [28,66,67].

Globally, these studies emphasise that NHPs such as TCM herbal formula, PUFAs and probiotics may influence the onset and severity of FPs-related gastrointestinal AEs in patients with GI. These beneficial effects can be attributed mainly to their anti-inflammatory and immunomodulatory properties.

In contrast, amino acid supplementation does not appear to have significant protective effects. L-cysteine and L-theanine have only shown a tendency to reduce the incidence of diarrhoea [15], unlike glutamine [16] for which clinical studies have not confirmed the promising results obtained in vivo suggesting a glutamine-associated preventive effect, mainly against radiotherapy-induced gastrointestinal toxicity [58,59].

### 3.2. NHPs and FP-Related Hematological Toxicity

Myelosuppression is one of the main FP-related toxicities [68,69]. Similar to FP-related gastrointestinal AEs, most RCTs and observational studies have focused on NHPs belonging to TCM [70]. Among them, *Curcuma wenyujin* containing β-elemene, which is used for the treatment of various malignancies [71], has been suggested to increase the efficacy and reduce toxicity of chemotherapy through multiple pathways [29,71]. In gastric cancer (GC) patients, Chen et al. demonstrated that hyperthermic intraperitoneal chemotherapy with CAPOX combined with β-elemene lowered the severity of myelosuppression compared with the control group not taking this NHP (*p* = 0.014) [29]. Jia Ru et al. reported that patients with stage III CRC undergoing adjuvant therapy with FPs combined with PRM1201 showed a lower incidence of grade 3 or 4 neutropenia, although this did not reach statistical significance [32]. In the RCT by Sun et al. involving patients with stage II-III CRC undergoing CAPOX therapy, treatment with JPBS reduced the incidence of grade ≥ 2 leukaemia and anaemia but increased the incidence of grade ≥ 2 thrombocytopenia compared with placebo (*p* = 0.012) [34]. The inhibition of the myelosuppressive effect has been attributed to icariin, an active compound present in JPBS that has been shown in vivo to stimulate the migration and proliferation of BMSC through the activation of the PI3K/AKT/mTOR pathway and inhibition of the ERK1/2 and JNK pathways [72]. Kong et al. demonstrated that the use of the Fuzheng Jiedu Quyu (FJQR) formula in combination with CAP-based chemotherapy in a cohort of 129 patients with HER-2-negative GC reduced the incidence of grade 3–4 ADRs, including leukopenia, anaemia, and thrombocytopenia, compared with placebo (*p* < 0.05) [26].

In a retrospective study, Yao et al. demonstrated that patients with stage II-III GC treated with another TCM NPH, Shengbai decoction (SBD), in combination with FP-based adjuvant chemotherapy had significantly fewer myelosuppression events, such as neutropenia (*p* = 0.04), thrombocytopenia (*p* = 0.03), and anemia (*p* = 0.04) [38].

Coix Seed Oil (Kanglaite^®^), another TCM compound, has been tested in the study by Zhan et al. [40]. The injection of this NHP with DOC chemotherapy regimen was useful in alleviating leukopenia and thrombocytopenia (*p* < 0.05) in a cohort of patients with GC [40]. This result was confirmed by a recent meta-analysis on CRC, which found that Coix injection plus chemotherapy (in particular FP-based treatment) resulted in a lowest incidence of leukopenia compared to injection of other NHPs belonging to TCM [73]. Other NHPs, in addition to those included in TCM, rich in vitamins, minerals, antioxidant enzymes and other bioactive compounds, have been studied as supportive agents in the management of various chronic conditions, including cancer [74]. In a prospective observational study by Avisar et al., 100 patients with stage II and III CRC undergoing adjuvant treatment with FPs were divided into an intervention group that received wheatgrass-juice (WGJ) and a control group. The intervention group had a significantly lower decline in white blood cell (WBC) count than the control group during chemotherapy [22].

Parenteral nutrition with Omegaven is recommended in critically ill patients, mainly due to its anti-inflammatory and antioxidant properties [15]. In patients with advanced oesophagogastric adenocarcinoma enrolled in a single-arm phase II study, weekly intravenous infusion of this NHP during palliative chemotherapy with epirubicin, OX and CAP (EOX) resulted in a significant reduction in thromboembolic events, but not in grade 3 or 4 neutropenia and leukopenia, compared to the group treated with EOX alone (0% vs. 19%, *p* = 0.04) [39].

Overall, these studies suggest that NHPs belonging to the TCM class may reduce myelosuppression in patients with CRC and GC. With regard to other NPHs, WGJ has been shown to significantly preserve immunological parameters, while Omegaven has been shown to be effective in reducing platelet aggregation, but without demonstrating benefits on other myeloid cells.

### 3.3. NHPs and Capecitabine-Associated Hand-Foot Syndrome

The management of CAP-induced Hand-Foot Syndrome (HFS) represents a significant clinical challenge in clinical practice, prompting growing interest in both preventive interventions and the identification of risk factors.

Hamaguchi et al. conducted a phase II, double-blind, placebo-controlled trial to evaluate the efficacy of oral L-cystine and L-theanine supplementation in preventing HFS in CRC patients undergoing CAP-based chemotherapy [15]. Preclinical data suggested that these amino acids act as precursors for glutathione (GSH) synthesis, thereby preserving redox balance and reducing oxidative stress in 5-FU–induced gastrointestinal injury models. However, as for gastrointestinal AEs, there was a non-statistically significant trend toward reduced incidence of grade ≥1 HFS (67.3% vs. 80.0%) in CRC patients receiving CAP when supplemented with L-cystine/theanine [75].

Another promising intervention is represented by the TCM compound, LC09. Based on preclinical studies, LC09 has anti-angiogenic and vasculoprotective roles, which could translate clinically into significant reductions in the severity of HFS. Yu et al. studied the effects of LC09 in 156 CRC patients undergoing CAP-based chemotherapy, demonstrating a significant improvement in HFS severity (*p* < 0.01) and pain reduction (*p* < 0.01), as well as a higher chemotherapy completion rate (*p* = 0.002) in patients treated with LC09 compared to the control group. These findings suggest that LC09 supplementation may have a beneficial impact on both CAP-related toxicity and treatment adherence [21]. Similarly, Zhao et al. in a multicentre, open-label study involving 92 CRC patients with various types of cancer treated with chemotherapy regimens including CAP, evaluated the efficacy of a modified Taohong Siwu decoction administered as a 30-min soak on the hands and feet. The authors reported a significantly higher clinical efficacy rate in the intervention group treated with NHP (88.3% vs. 50%; *p* = 0.0001), with marked improvements in pain, daily functioning, and overall QoL, as assessed by the HFS-14 scale [76]. The mechanism underlying these beneficial effects may be the modulation of the VEGF/HIF-1α pathway, as suggested by a preclinical model showing potential antiangiogenic effects resulting from the use of this TCM compound [77].

In contrast, in their double-blind RCT, Watanabe et al. found no significant benefit associated with the use of Kampo medicine Eppikajututo (TJ-28) in patients treated with CAP compared with pyridoxine (*p* = 0.114), indicating that there is no clear advantage to this intervention [18].

Contradictory results have also been reported on the use of topical formulations based on henna (*Lawsonia inermis* L.). Henna has traditionally been used for its anti-inflammatory, antimicrobial and antioxidant properties, largely attributed to its main active compound, lawsone (2-hydroxy-1,4-naphthoquinone) [78]. Although clinical evidence is limited, some preclinical studies have suggested potential cytoprotective and wound-healing effects of henna extracts in vitro and in animal models, indicating a possible role in mitigating skin toxicity [79]. Mohajerani et al. conducted a pilot randomised, double-blind, placebo-controlled trial involving 18 patients undergoing CAP or 5-FU chemotherapy with HFS to evaluate the potential beneficial effects of a topical henna hydrogel containing lawsone. The study found no statistically significant improvement in HFS severity compared to placebo, highlighting the need for larger-scale clinical trials to clarify its potential efficacy [17]. Elyasi et al. investigated the combined use of henna and curcumin (i.e., alpha ointment formulation) in a randomised, triple-blind, placebo-controlled study involving 110 CRC patients treated with XELOX+ Lapatinib. Although the ointment did not significantly prevent HFS, it was associated with a delay in the onset of this AE compared to placebo (*p* < 0.001), suggesting potential benefits [31]. Overall, these studies suggest a possible topical use of TCM compounds in the management of CAP-related HFS.

### 3.4. The Role of NHPs in Enhancing the Efficacy of FP-Based Therapy

In addition to potential safety benefits, there is evidence to suggest that NHPs may enhance the efficacy of FP-based therapy. Table 3 summarises the main characteristics of studies describing this potential ability.

In a RCT, Bagheri et al. demonstrated that a mediterranean diet based on extra virgin olive oil rich in monounsaturated fats and antioxidants such as polyphenols and vitamin E (EVOO) improved body composition and reduced systemic inflammation in CRC patients with cachexia undergoing FOLFOX, CAPOX or CAP regimen. Compared to the control group (CRC patients who received only dietary recommendations), after 8 weeks the intervention group showed a significant increase in body weight (*p* < 0.001), lean mass (*p* = 0.001), fat mass (*p* = 0.002), systemic inflammation (*p* < 0.001) and muscle strength (*p* < 0.001), as well as significant reductions in serum levels of TNF-α (*p* < 0.001), IL-6 (*p* < 0.001) and C-reactive protein (*p* = 0.01) levels, and improvements in overall health and physical function scores (*p* = 0.02 and *p* < 0.001, respectively) [14].

During four cycles of chemotherapy, patients in the placebo group experienced a significant worsening of fatigue and QoL scores (both *p* = 0.02), whereas this was not observed in the group taking zinc supplements, indicating a protective effect of this trace element against the progression of chemotherapy-induced fatigue [80].

In a multicenter RCT, Wu et al. reported that adding the Hezhong granules to standard antiemetic therapy in patients with advanced CRC receiving FPs (CAPEOX or mFOLFOX6 or FOLFIRI or CAP) significantly delayed CINV. Specifically, the rates of CRR and ORR increased by 20% and 18% (*p* = 0.035 and *p* = 0.043, respectively), and the mean number of daily CINV events decreased by 19% (*p* < 0.05) compared to placebo. In addition, a significant improvement (*p* = 0.004) was observed in the Functional Living Index–Emesis (FLIE) score, a validated questionnaire that assesses the impact of nausea and vomiting on QoL [19]. In a RCT examining the effects of daily WGJ administration during adjuvant chemotherapy (CAP alone or CAP + OX or 5-FU/LV + OX), analysis of extracellular-vesicles (EVs) showed lower levels of thrombogenic markers (tissue-factor levels, *p* = 0.029; endothelial protein C receptor, *p* = 0.005) in the WGJ group compared to the control group treated with chemotherapy alone [22]. Jia et al. demonstrated that adding the PRM1201 formulation to adjuvant FOLFOX or CAPOX regimens for 6 months in patients with stage III CRC significantly improved DFS. Specifically, the DFS was 91.2% after 1 year, then decreased to 84.5% and 77.1% after 2 and 3 years, respectively, in the group treated with chemotherapy+PRM1201, compared to 89.7%, 79.2% and 68.6%, respectively, after 1, 2 and 3 years in the arm treated with chemotherapy+placebo (*p* = 0.024) [32].

In a multicenter RCT, Sun et al. evaluated the effect of the herbal formula JPBS on the completion of adjuvant chemotherapy with CAPOX in patients with stage II–III CRC. Successful completion of chemotherapy cycles was significantly higher in the JPBS group than in the placebo group (*p* = 0.003) with improved completion rates in stage II patients (*p* = 0.001) and younger patients (*p* = 0.004) without compromising tolerability [34].

Zhao et al. investigated the effect of Taohong Siwu decoction in patients with CAP-associated HFS. Compared to the control group, patients receiving the decoction had a significantly higher efficacy rate (efficacy refers to the ability to reduce pain, ulcers, and muscle atrophy, even if the patient continued to experience pain in the extremities) (83.3% vs. 56.7%, *p* < 0.05). The study suggests that this traditional formula provides clinically meaningful relief of chemotherapy-related dermatological complications [76].

Eltweri et al. performed a phase II clinical trial to evaluate the impact of Omegaven^®^ supplementation in patients with advanced esophagogastric adenocarcinoma receiving epirubicin, OX and CAP. Patients supplemented with Omegaven^®^ achieved significantly improved ORR (45% vs. 23%, *p* = 0.04) and longer median PFS (7.1 vs. 4.7 months, *p* = 0.02) compared with controls [39].

Kong et al. reported that FJQR plus CAP in GC patients significantly prolonged PFS compared with FP alone (median 7.3 vs. 5.1 months, *p* < 0.05). The findings support the synergistic efficacy of FJQR in maintaining treatment benefit after chemotherapy [26].

Verma et al. conducted a single-arm phase II trial to test YIV-906 in combination with CAP neoadjuvant chemotherapy and radiotherapy in locally advanced rectal cancer. At a median follow-up of 61.9 months, the mean survival of the patient-based cohort was 74.9 months [95% CI: 67.3–82.5]. The predicted 3- and 5-year OS rates were 91.2% and 82.0%, respectively. The median PFS was 58.3 months (95% CI: 47.8–68.8). The estimated 3-year and 5-year PFS rates were 74.6% and 58.5%, respectively [27].

CAPOX chemotherapy in GC patients, combined with Zhipu Liuyunzi decoction, led to a marked improvement in clinical efficacy, with higher rates of objective remission and disease control (*p* < 0.05); reduced tumour marker levels (*p* < 0.001); higher levels of immune parameters (*p* < 0.001) and QoL (*p* < 0.001) compared to chemotherapy alone. These results indicate an enhanced therapeutic effect when the decoction is combined with standard treatment [37].

Yao et al. conducted a retrospective analysis to evaluate the survival of Shengbai decoction in GC patients undergoing CAPOX- and FOLFOX treatment. Patients treated with the herbal formula showed significantly longer median OS (46.7 vs. 39.2 months, *p* < 0.05) compared with controls [38]. Zhao et al. conducted a multicenter randomised controlled trial to evaluate the efficacy of Jianpi Jiedu decoction in patients with stage II-III CRC who underwent radical resection and were treated with DOC regimen. After three years, the intervention group showed a significantly lower recurrence and metastasis rate than the control group (16.7% vs. 27.4%, *p* = 0.02), along with improved DFS [76].

The studies described above demonstrate that there is significant evidence of improved efficacy outcomes, particularly with NHPs belonging to TCM. PRM1201 has been shown to improve DFS when added to FOLFOX or CAPOX regimens in stage III CRC [32], while JPBS [34] and LC09 [21] increase chemotherapy completion rates, particularly in patients with stage II CRC and younger subjects. Notably, YIV-906 administered with neoadjuvant chemoradiotherapy in CRC has shown encouraging long-term survival rates, with 3- and 5-year OS exceeding 90% and 80%, respectively, and durable PFS [27].

### 3.5. The Effects of NHPs Combined with Folate Supplements

Unlike the studies mentioned above, in which NHPs demonstrated protective or neutral effects on the onset and severity of FP-related AEs, the role of combining folate supplements and FP should be contextualised based on various risk factors. The addition of folate (folic acid and its synthetic derivative, folinic acid) during chemotherapy raises the question of the delicate balance between safety and efficacy. In fact, folate is essential for the human body, but it can interfere with certain chemotherapy drugs, particularly antifolates and FPs, and excessive or insufficient folate intake can affect the efficacy and tolerability of chemotherapy treatment [81].

For example, when pemetrexed is administered, folinic acid is used to reduce its toxicity, “saving” healthy cells [82].

Conversely, higher folate levels have been associated with an increased risk of FP-related toxicity. This is because folic acid and folinic acid are very similar in terms of structure-activity relationship, and both, in their reduced form, inhibit TS. For this reason, concomitant intake of these molecules may lead to an increased risk of FP-related AEs, particularly nausea and vomiting, HFS, diarrhoea, and neutropenia [23]. Therefore, patients should only take specific folate supplements under the guidance of an oncologist [83].

The explanation for this important issue is that folate intake through diet and supplementation can interfere with MTHFR activity, improving the efficacy of FPs but also increasing the severity of FP-related AEs [84].

In this context, we previously reported the clinical case of two patients treated with CAP or CAPOX at a dosage greater than 2000 mg/m^2^ in combination with folic acid who experienced serious FP-related toxicity (grade 3 diarrhoea and grade 4 HFS) [10]. According to the summary of product (SmPC) of CAP, 2000 mg/m^2^ per day is the maximum tolerated dose (MTD) when administered concomitantly with folinic or folic acid [1]. Both patients were taking folic acid during chemotherapy without informing their oncologists. Furthermore, both patients were homozygous for the TSER 2R/2R genotype, a polymorphic variant in the promoter enhancer region of the TYMS gene encoding TS, which has been proposed as a predictor of CAP-related toxicity, suggesting the involvement of genetic and non-genetic factors in determining CAP-related toxicity [85,86].

The results of other studies reviewed here confirm that concomitant administration of CAP and folate may increase the toxicity of chemotherapy.

The study conducted by King et al., aimed to investigate the risk factors involved in the onset of HFS of any grade and HFS of grade ≥ 2 in the Malaysian cancer population undergoing CAP-based treatment. Of the 369 patients enrolled, approximately half developed HFS of any grade, of which 14.6% were grade ≥ 2. The risk factors most associated with the onset of HFS of any grade were higher doses of CAP (≥2500 mg/m^2^/day) (OR 2.96, 95% CI 1.62, 5.38) and folic acid intake through both diet and supplements (OR 3.27, 95% CI 1.45, 7.35) [24]. Therefore, CAP dose intensity and folic acid intake emerged as significant predictors of more severe HFS, with folic acid intake associated with increase in grade ≥ 2 HFS although without reaching a statistical significance.

Furthermore, Kawakita et al. investigated the potential correlation between folate intake and MTHFR and TYMS polymorphisms. In particular, MTHFR C677T and a 6 bp deletion (del) in the 3′-untranslated region of TYMS were analysed. TYMS 1494del6 was found to be associated with reduced mRNA stability and lower TS expression [87,88]. Four hundred and thirty-seven patients with head and neck squamous cell carcinoma (HNSCC), undergoing 5-FU chemotherapy were enrolled in this study and stratified, according to dosage and frequency of folic acid intake, in three groups: low (240 μg/day), medium (>240 and <320 μg/day) and high (320 μg/day). Some patients (16%) also took vitamin B9 supplements compared to 76% who did not. Patients with high folate intake had better OS and DFS than patients with low and medium folate intake (*p* = 0.006). Patients with TYMS polymorphism showed worse OS than the wild type (*p* = 0.093). No significant correlation was found between folate intake and MTHFR polymorphism with OS or DFS [89].

Similarly, Shitara et al. attempted to understand whether the abovementioned MTHFR and TYMS gene polymorphisms could affect efficacy and safety outcomes in patients taking foods and supplements containing folic acid and undergoing FP-based chemotherapy. In this study, 132 advanced GC patients treated with 5-FU-based therapy were retrospectively analyzed. The patients with a folate intake > 260 micrograms/day had longer OS than those with a folate intake ≤ 260 μg/d (12.2 months vs. 8.4 months, *p* = 0.03). It was observed that patients with a folate intake > 260 microg/day, homozygous for genetic variants MTHFR 677TT (*p* = 0.039) and wild type for TYMS 1494del6 (*p* = 0.01) had a better OS. A similar trend was observed for PFS, however without achieving statistical significance (*p* = 0.094). Conversely, no association was found between the presence of TYMS-2R/2R genotype and OS or PFS. However, it is important to note that the incidence of grade 3–4 haematological and non-haematological toxicity was significantly higher in patients with moderate/high folate intake compared to those with low intake, after adjusting for age, performance status, gender and treatment regimens. In addition, MTHFR 677TT tends to be associated with a higher frequency of haematological toxicity, although this did not reach statistical significance [90].

About the correlation between folic acid intake and the onset and severity of AEs, factors other than pharmacogenetics such as serum and red cell folate concentration, should be evaluated. As red blood cells (RBCs) sequester approximately 95% of folate, measuring folate concentration in RBCs is useful to evaluate the individual capability to store these elements and so it can be considered a reliable indicator of long-term folate stores while serum folate reflects recent dietary intake and provides an earlier indication of deficiency [91]. In a prospective observational study, Chan et al. measured serum and RBC folate concentrations in 40 and 86 CRC patients prior to treatment with CAP and CAPOX, respectively. Overall, 80 patients (63.5%) experienced grade 2 toxicity, while 18 patients (14.3%) experienced grade 3 toxicity. The most common grade 2 AEs were CINV (47.7%), HFS (25.4%), diarrhoea (23.1%), and neutropenia (22.3%). Grade 2 toxicity was more frequent in patients with higher serum folate levels, with the risk increasing by 9% for every 10 nmol/L rise. Patients experiencing grade 2 toxicity had significantly higher serum folate levels compared to those with grade 1 toxicity (*p* = 0.001). Serum folate levels were particularly associated with skin toxicity as well as nausea and vomiting. Notably, 76.1% of patients who did not receive folate-containing supplements exhibited toxicity, whereas 100% of patients who received folate-containing dietary supplements developed it. However, serum and RBC folate levels did not differ significantly between patients who received supplements and those who did not [23].

In the “COLON” prospective observational study, 290 patients with stage II-III CRC treated with CAP, dietary and supplemental folate and folic acid intake were assessed at diagnosis and during chemotherapy. At the same time, folate and folic acid plasma levels were measured. The results showed that the use of folic acid supplements during treatment (hazard ratio (HR) 1.81 and 95% confidence interval (CI) 1.15–2.85) and the presence of circulating folic acid at diagnosis (HR 2.09, 95% CI: 1.24, 3.52) and during treatment (HR 2.31, 95% CI: 1.29, 4.13) were associated with an increased risk of CAP-related toxicities. Dietary folate intake or plasma folate levels stand-alone were not associated with the risk of these toxicities [25].

Similarly, in a randomised phase II trial including 294 GI patients treated with CAP, Yap et al. concluded that high folate levels in serum (OR, 1.30; 95% CI, 1.12–1.52; *p* < 0.001) and RBCs (OR, 1.28; 95% CI, 1.10–1.49; *p* = 0.001) were associated with an increased risk of grade ≥ 2 HFS. In addition, grade 2 or higher HFS was linked to 300 DNA variants with significant genomic significance, including a new DPYD variant (rs75267292, *p* < 0.0001) and variants in MACF1 (Microtubule Actin Crosslinking Factor 1) (rs183324967, *p* < 0.0001 and rs148221738, *p* < 0.0001) and SPRY2 (Sprouty 2) (rs117876855, *p* < 0.0001; rs139544515, *p* < 0.0001) involved in the pathways of tissue repair [20].

The results of the studies reviewed here confirm that the use of folate supplements or folate dietary intake may contribute to FP-related ADRs, such as diarrhoea and HFS but also to an improvement of FP efficacy.

**Table 3:** Main characteristics and results of RCTs and observational studies investigating the efficacy of NHPs in cancer patients treated with fluoropyrimidine-based chemotherapy.

Study Design	Country	Patients (n)	Type of Cancer	Sex (F%)	Median Age and Age Range (Years)	Food Supplements	Concomitant Drugs	Follow-Up	Efficacy Outcomes	Main Results and Conclusion	Jadad Scale or NOS	Ref.
RANDOMIZED CONTROLLED TRIAL-EFFICACY												
RCT	Iran	46 INT-g: 23 CT-g: 23	CRC	30.5	58.3 (48–68)	Mediterranean diet regime with EVOO	FOLFOX or CAPOX or CAP	NA	Valuation of muscle strength, lean body mass, nutritional status, inflammatory markers, QOL, serum albumin and total protein	A significant increase in weight, lean mass, fat mass, fat percentage, muscle strength and total serum protein was found in the INT-g compared to the CT-g (*p* < 0.001). In addition, there was a significant reduction in TNF-α, Hs-CRP and IL-6 in the INT-g group compared to the CT-g group (*p* < 0.001).	3	[14]
RCT	China	52 pz INT-g: 27 CT-g: 25	GC	41	69.3 (65–75)	Elemene	Lobaplatin perfusion (50 mg/m^2^), CAPOX	9.04 ± 4.30 (3.00–17.90)	OS	No OS benefit was observed in the INT-g. Elemene can decrease the occurrence of myelosuppression in metastatic GC.	2	[29]
Randomized, placebo-controlled, triple-blind clinical trial	Brazil	56 INT-g: 28 CT-g: 28	GI	47	54.5 (41–63)	EPA and DHA 1.55 gr/day	CAPOX or OX + FU or FU+LV	6 and 12 months	Treatment response, performance status	Fish oil can result in a better performance status for GC patients undergoing chemotherapy. No treatment response difference was found between groups.	5	[30]
Randomised double-blinded, placebo-controlled clinical trial	China	370 INT-g: 186 CT-g:184	CRC	42.7	60.3 (32–75)	PRM1201 twice a day for 6 months	FOLFOX or CAPOX	29.60 months	DFS, QOL	An increase in the 3-year DFS rate (*p* = 0.024) and QoL (*p* < 0.05) was observed in the INT-g compared to CT-g.	5	[32]
Placebo-Controlled, Prospective Randomised Trial	Brazil	24 INT-g: 14 CT-g: 10	CRC	62.5	63.2 (50–75)	Zinc sulfate, 154 mg (corresponding to 35 mg of elemental zinc), two capsule	CAPOX or CAP or 5-FU/LV	4 months	Impact of zinc supplementation on antioxidant enzymes, vitamins, and lipid peroxidation during chemotherapy cycles	Patients treated with zinc showed increased SOD activity and reduced GPx activity compared to those treated with placebo (*p* < 0.05). Vitamin E levels remained stable in the INT group, while they decreased in the CT group (*p* < 0.05). Furthermore, zinc supplementation did not affect lipid peroxidation (*p* < 0.05).	3	[80]
Multi-center, phase III, randomized, placebo-controlled trial	China	400 INT-g: 200 CT-g: 200	CRC	43.1	60.3 (60–80)	JPBS	CAPOX	NA	Chemotherapy completion rate, QOL	Successful completion of chemotherapy cycles was significantly higher in the INT-g than in the CT-g (*p* = 0.003), with improved completion rates in stage II patients (*p* = 0.001) and younger patients (*p* = 0.004), without compromising tolerability. QOL also improved in stage II patients and younger patients.	5	[34]
Double-blind, randomized, controlled trial	China	92 INT-g: 60 CT-g: 32	CRC	41.9	NA (36–78)	Taohongsiwu Decoction (for external Application) (*Semen Persicae*, *Flos Carthami*, *Radix Rehmanniae Praeparata*, *Radix Angelicae Sinensis*, *Rhizoma Chuanxiong*, *Radix Paeoniae Alba*, *Ramulus Cinnamomi*, *Radix Cyathula*, *Radix Glycyrrhizae*, and *Jujubae* >39 °C decoction during soaking on hands and feet for 30 min, once a day, for 30 min each time, for 7 days.	CAP, SF, GEF	3 months	Effectiveness and QoL	A significant difference was observed in the effective rate of the INT-g, which was higher (88.3%) than the 50% of the CT-g (*p* = 0.00). Modified Taohongsiwu decoction is effective in treating patients with HFS and enhances their QoL, as measured by the HFS-14.	5	[76]
Multicenter, randomized, double-blind placebo-controlled clinical trial	China	112 INT-g: 56 PL-g: 56	CRC	39.3	63.1 (52–74)	Hezhong granules 6 g three times daily, for a total daily dose of 18 g from days 1–14 of chemotherapy, followed by a 7-day break.	FU-based chemotherapy (CAPEOX, mFOLFOX6, FOLFIRI, and CAP monotherapy (plus 5-HT3-receptor antagonist, DXM)	NA	Efficacy and safety in the prevention of CINV (chemotherapy- induced nausea and vomiting)	In the delayed phase, significant improvements in INT-g were observed in both CRR (*p* = 0.053; *p* = 0.035) and ORR (*p* = 0.037; *p* = 0.043) for nausea and vomiting. Additionally, the daily frequency of CINV events showed a mean difference of 19% (*p* < 0.05). No serious adverse events were attributed to phytotherapy.	4	[19]
Randomized, Multiple-center, double-blind, and parallel-controlled trial	China	156 INT-g: 78 CT-g: 78	CRC	70.4	55.5 (38–72)	Chinese herbal compound LC09 110 gr per day	CAP-containing chemotherapy for 2 weeks, followed by a 1-week break	1 month and 3 months after completing the trial	Chemotherapy completion rate	The chemotherapy completion rate in the INT-g group (62.86%) was significantly higher than that in the CT-g group (40.28%), *p* = 0.0021.	5	[21]
OBSERVATIONAL STUDIES—EFFICACY												
Not Randomized clinical trial (volunteers bias)	Israel	CRC	100 INT-g: 50 CT-g: 50	44.4	64 (29–83)	WGJ	1. CAP alone 2. CAP + OX 3. 5-FU/LV + OX	NA	Difference in chemotherapic effects	The INT group showed a reduction in vascular damage and chemotherapy-related thrombogenicity (tissue factor levels *p* = 0.029 and endothelial protein C receptor *p* = 0.005), growth factors, cytokines (*p* = 0.0001), endothelial VEs (*p* = 0.0015) compared to the CT group.	6	[22]
Phase II single-arm clinical trial with historical CT-g group	United Kingdom	GC	58 INT-g: 21 Historical CT-g: 37	27.6	66.5 (36–81)	Omega-3 fatty acids (Omegaven^®^) once a week for a 4 h infusion, once a week	EOX (Epirubicin 50 mg/m^2^ and OX 130 mg/m^2^ every 21 days and oral CAP)	3 months following completion of treatment	Improvement of radiological response	Partial response (*p* = 0.03) was higher in the INT-g than in the CT-g. However, no statistically significant difference in stable disease, progressive disease, OS and PFS was found between the two groups. Significant reductions in IL-2, TNF-α and VEGF concentrations were observed (*p* = 0.009, *p* < 0.0001, *p* = 0.002, respectively) after each treatment.	06	[39]
Retrospective study	Japan	HNSCC	437 low folate intake-g: 135 medium folate intake-g: 143 high folate intake-g: 144 unknown: 15	22	61 (21–79)	Dietary folate intake (low/medium < 320 μg/day; high ≥ 320 μg/day)	CDDP + 5-FU NDP+ 5-FU CDDP alone	4.9 years	Association between dietary folate intake and MTHFR and TYMS polymorphisms with OS and DFS in HNSCC.	Patients with high folate intake had significantly higher survival than patients with low or average folate intake (*p* = 0.020). No significant association was found between MTHFR and TYMS polymorphisms and survival.	7	[89]
Retrospective study	China	GC	129 INT-g: 64 CT-g: 65	28.7	NA (18–75)	“Fuzheng jiedu Quyu Method” recipe (FJQR) 15 g Bid	CAP-based treatment (CAP 1000 mg/m 2 per os, Qid)	<1 month	Evaluate efficacy of FJQR combined with CAP-based chemotherapy	The mPFS in the INT-g was significantly higher than the CT-g group (6.3 vs. 5.0 months, *p* = 0.03).	7	[26]
Retrospective study	Japan	GC	132 Low folate intake group: 44 Medium folate intake group: 44 High folate intake group: 44	31	58 (30–80)	Dietary folate intake low (≤260 μg/d), medium (>260 and ≤340 μg/d), high (>340 μg/d)	S1+CDDP S1+docetaxel S1+irinotecan 5-FU+CDDP 5-FU+methotorexate	NA	Assess role of dietary folate intake and polymorphisms on OS and PFS in gastric cancer	Patients with folate intake > 260 μg/day, carrying the MTHFR 677 TT polymorphism and TYMS-3′ untranslated region 6-bp insertion had significantly better OS (*p* = 0.030; 0.039; 0.01). Better PFS was significantly associated with the presence of the MTHFR 677 TT polymorphism (*p* = 0.046) and the insertion of the 6-bp untranslated region of TYMS-3′ (*p* = 0.029).	8	[90]
A single-arm phase II clinical trial with historical cohort	USA	CRC	24	25	54.5 (50–62)	YIV-906 800 mg bid on days 1–4 of RT each week	CAP (orally at a dose of 825 mg/m 2 bid, on days 1–5 of RT (pelvic radiation therapy) each week or FOLFIRI or CAPOX or FOLFOX	61.9 months	OS, PFS	The 3- and 5-year overall survival rates were 91.2% and 82.0%, respectively. The mean progression-free survival was 58.3 months (95% CI: 47.8–68.8), with corresponding 3- and 5-year rates of 74.6% and 58.5%.	6	[27]
Retrospective study	China	GC	100 INT-g: 50 CT-g: 50	51	55.5 (48–63)	Zhipu Liujunzi decoction	OX 130 mg/m^2^ e.v b.i.d once a day and orally taking 1500 mg of CAP b.i.d for consecutive 14 day	1 month	Evaluate the clinical efficacy of co-administration of Zhipu Liujunzi decoction with CAPOX treatment	Patients in the INT group showed significantly greater cancer resistance, lower tumour marker levels (*p* < 0.001); higher rates of objective remission and disease control (*p* < 0.05); better immune parameters (*p* < 0.001) and QOL, (*p* < 0.001).	6	[37]
Retrospective study	China	GC	115 INT-g: 57 CT-g: 58	31	54.8 (48–62)	Shengbai decoction 6 g, 100 mL twice a day half an hour before meals	SOX, CAPOX, FOLFOX	Every 3 months	Evaluate effect of Shengbai decoction on long-term survival	Patients in the INT-g showed significantly better 3-year RFS (*p* = 0.0369) and 3-year OS (*p* = 0.0455) compared to CT-g.	7	[38]
Clinical trial	China	GC	60 INT-g: 30 CT-g: 30	NA	NS (18–75)	Kanglaite^®^ 100 mL intravenous per day during chemotherapy	DOC	Every three weeks	Efficacy of Kanglaite^®^ injection	The response rate and KPS score in the INT-g improved compared to those in the CT-g (*p* < 0.05). In addition, gastrointestinal and haematological adverse reactions were lower than in the CT-g (*p* < 0.05).	5	[40]

Abbreviations: B, bevacizumab; CAP, capecitabine; CAPOX, capecitabine/oxaliplatin; CDDP, Cisplatin; CRC, colorectal cancer; CRR, the proportion of patients without nausea/vomiting; DFS, Disease-free survival; DHA, docosahexaenoic acid; DOC, Docetaxel/Oxaliplatin/Capecitabine; DXM, dexametasone; EOX, Epirubicin/Oxaliplatin; EPA, eicosapentaenoic acid; EVs, Extracellular vesicles; EVOO, extra virgin olive oil; FJQR, Fuzheng jiedu Quyu Method Recipe; FOLFIRI, fluorouracil/leucovorin/irinotecan; FOLFOX, fluorouracil/leucovorin/oxaliplatin; FU, fluorouracil; FU/LV, fluorouracil/leucovorin; GC, gastric cancer; GI, gastrointestinal cancer; GEF, gefitinib; GPx, glutathione peroxidase; HFS, Hand-foot syndrome; HCC, Hepatocellular carcinoma; HNSCC, Neck squamous cell carcinoma; HS-CRP, High Sensitivity C-reactive Protein; IL-6, Interleukin; JPBS JianPi-Bushen Formula; LAP, Lapatinib; LV, leucovorin; MTHFR, Methylenetetrahydrofolate reductase; NDP, nedaplatin; NOS, Newcastle–Ottawa scale; NYT, Ninjin’yoeito; OX, oxaliplatin; SF, Sorafenib; ONS, Oral Nutritional Supplements; ORR, the proportion of patients without nausea/vomiting plus mild nausea/vomiting; OS, Overall Survival; QOL, quality of life; RDI, relative dose intensity; Ref., References; RFS, recurrence-free survival; S-1, Tegafur, 5-Chloro-2-4-Dihydroxypyridine and Oxonic Acid; SOD, Superoxide Dismutase; SOX, Oxaliplatin combined with S-1; TYMS, Thymidylate Synthetase gene; TJ-14, Hangeshashinto; TNF-ɑ, Tumor Necrosis Factor-alpha; VEGF, Vascular endothelial growth factor; KPS, Karnofsky Performance Status; WGJ, wheatgrass juice; XELOX, Capecitabine/Oxaliplatin.

Most of the studies have demonstrated that high folate intake [23,24,25,90], as well as their high concentrations in serum [20,23], plasma [25], and RBCs [20,23] may be associated with an exacerbation of ADRs typically related to FPs, mainly HFS [20,23,24], diarrhoea [23], myelosuppression [23,90] and CINV [23]. In fact, folate availability, although physiologically important, when present at excessively high levels, especially in the presence of other risk factors, may increase susceptibility to CAP-induced toxicity. In particular, polymorphisms in genes involved in the FP pathway, including MTHFR and TYMS polymorphisms have been associated with a higher incidence of FP-related ADRs [89,90].

Moreover, the amount of folate intake may be correlated with FP efficacy. In fact, some authors have reported that patients with GI or head neck cancer with high folate intake had better OS [89,90], PFS [90] and DFS [89] than patients with medium and low folate intake. Genetic polymorphisms also play a role in terms of efficacy. In fact, Shitara et al. have observed that patients who are homozygous for MTHFR C677T and wild type for TYMS 1494del6 had better OS and PFS [90]. Similarly, Kawakita et al. have shown that patients who were homozygous for TYMS 1494del6 had worse OS than wild-type patients [89]. Unfortunately, not all studies evaluated these variables together, preventing the possibility of estimating the impact of each of them on the effects of the folate-FP combination.

## 4. Conclusions

This updated review suggests that NHPs may be useful in managing FP-related toxicity. The most important findings concern TCM compounds, including Kanglaite^®^, which has been suggested to improve clinical response and reduce gastrointestinal and hematological toxicity in CG patients, while topical administration of various decoctions has been suggested to be useful in managing CAP-related HFS and FP-related gastrointestinal AEs. In addition, some TCM compounds, as well as the omega-3 fatty acids EPA and DHA, have been recognised as capable of reducing toxicity and improving the efficacy of FP-based chemotherapy. TCM compounds are complex mixtures of multiple herbs with numerous active ingredients. This makes it difficult to identify which components are responsible for beneficial or AEs. Obtaining high-quality scientific evidence is challenging, mainly because active ingredients in TCM compounds are not well characterized in terms of pharmacokinetics and pharmacodynamics, and potential interactions with conventional therapies. Most traditional formulas are studied as a whole, highlighting the need for research on individual herbs to clarify their pharmacological profiles. Among the products described in this review, only Omegaven^®^, Kangliate^®^ and Kefbios^®^ are registered and standardized, ensuring consistent quality. Based on the available literature, folate supplements can improve the efficacy of FP-based chemotherapy, but they can also cause an increase in toxicity. This depends on genetic and non-genetic factors, such as comorbidity and the use of multiple drugs. However, in all the studies reviewed, no reference was found to comorbidity or polypharmacy in patients undergoing chemotherapy. This is a major concern, as folate is often used by cancer patients without the knowledge of healthcare professionals. Before considering adding NHPs to clinical practice, large-scale RCTs are needed that integrate congenital and environmental variables specific to each individual patient.

## Data Availability

No new data were created or analyzed in this study.
